# Systolic Blood Pressure Response to Exercise in Endurance Athletes in Relation to Oxygen Uptake, Work Rate and Normative Values

**DOI:** 10.3390/jcdd9070227

**Published:** 2022-07-15

**Authors:** Anna Carlén, Gustaf Eklund, August Andersson, Carl-Johan Carlhäll, Magnus Ekström, Kristofer Hedman

**Affiliations:** 1 Department of Clinical Physiology in Linkoping, and Department of Health, Medicine and Caring Sciences, Linkoping University, 58183 Linkoping, Sweden; gusek496@student.liu.se (G.E.); augan314@student.liu.se (A.A.); carl-johan.carlhall@liu.se (C.-J.C.); kristofer.hedman@liu.se (K.H.); 2 Department of Respiratory Medicine, Allergology and Palliative Medicine, Lund University, 22185 Lund, Sweden; magnus.ekstrom@med.lu.se

**Keywords:** blood pressure, exercise, work rate, oxygen uptake, endurance athletes, SBP/W slope, SBP/VO_2_ slope

## Abstract

Work rate has a direct impact on the systolic blood pressure (SBP) during aerobic exercise, which may be challenging in the evaluation of the SBP response in athletes reaching high work rates. We aimed to investigate the exercise SBP response in endurance athletes in relation to oxygen uptake (VO_2_), work rate and to recent reference equations for exercise SBP in the general population. Endurance athletes with a left-ventricular end-diastolic diameter above the reference one performed a maximal bicycle cardiopulmonary exercise test. The increase in SBP during exercise was divided by the increase in VO_2_ (SBP/VO_2_ slope) and in Watts, respectively (SBP/W slope). The maximum SBP (SBP_max_) and the SBP/W slope were compared to the predicted values. In total, 27 athletes (59% men) were included; mean age, 40 ± 10 years; mean VO_2max_, 50 ± 5 mL/kg/min. The mean SBP/VO_2_ slope was 29.8 ± 10.2 mm Hg/L/min, and the mean SBP/W slope was 0.27 ± 0.08 mm Hg/W. Compared to the predicted normative values, athletes had, on average, a 12.2 ± 17.6 mm Hg higher SBP_max_ and a 0.12 ± 0.08 mm Hg/W less steep SBP/W slope (*p* < 0.01 and *p* < 0.001, respectively). In conclusion, the higher SBP_max_ values and the less steep SBP/W slope highlight the importance of considering work rate when interpreting the SBP response in endurance athletes and suggest a need for specific normative values in athletes to help clinicians distinguish physiologically high maximal blood pressure from a pathological blood pressure response.

## 1. Introduction

An exaggerated blood pressure response to exercise has been defined in guidelines as a maximum systolic blood pressure (SBP_max_) of at least 210 mm Hg in men or 190 mm Hg in women [1,2]. However, these thresholds fail to account not only for age, but also for the relation between work rate and SBP, which is physiologically important as the mean arterial pressure (MAP) is estimated as the product of cardiac output (CO) and the total peripheral resistance. Thus, a high work rate typically yields a high SBP through increased CO [3], which, in turn, is closely related to high oxygen uptake (VO_2_) [4]. This makes interpretation of the SBP response to exercise particularly challenging in athletes, who often reach work rates far exceeding those of the general population and, therefore, may be expected to present with a higher SBP_max_. Although peak exercise blood pressure above a preset threshold both in the general population [5] and in athletes [6] is associated with an increased risk of future hypertension, the impact of exercise capacity on blood pressure response and subsequent cardiovascular risk in athletes is scarcely studied. 

Indexing the increase in SBP to the increase in work rate has gained recent attention as a way of accounting for the effect of work rate on the SBP_max_ [7,8]. A reference equation for the SBP/W slope for the general population was recently published [9]. Furthermore, for the increase in SBP in relation to the increase in metabolic equivalents of task (MET), the SBP/MET slope, upper limits have been suggested [7]. In addition, a few recent studies have investigated the SBP/W and the SBP/MET slopes in young athletes engaged in team sports [10,11]. There is, however, a lack of studies specifically dedicated to endurance athletes, who through their enhanced aerobic capacity can reach the highest VO_2_ values and, thus, may be expected to reach even higher SBP_max_ values. Evaluation of the SBP increase in relation to the direct measurement of VO_2_ has also been suggested in athletes, providing the SBP/VO_2_ slope [12].

The primary aim of this study was to characterize the SBP response to exercise in male and female endurance athletes, including the SBP/VO_2_ and the SBP/W slopes, and to compare the SBP_max_ and the SBP/W slope to the values predicted by recent reference equations [9]. 

## 2. Materials and Methods

In this explorative cross-sectional study, data were collected at the Department of Clinical Physiology at Linköping University Hospital, Linköping, Sweden, between January 2020 and February 2021. 

Endurance athletes were recruited through a general advertisement to undergo a brief screening echocardiographic examination (Vivid E95 cardiac ultrasound, GE Healthcare, Chicago, IL, USA) for eligibility. Athletes were considered for inclusion if they had (a) a left-ventricular end-diastolic diameter measured in the parasternal long-axis view above the body size-indexed age- and sex specific upper limit of normal [13], (b) no cardiac pathology (absence of structural heart disease and normal systolic left ventricular function [14]), (c) no prior cardiovascular disease and (d) no prior cardiotoxic chemotherapy. The rationale for studying athletes with a large left ventricle was to assure that only subjects with a sufficient dose of endurance exercise to elicit left ventricular adaptations were included [15,16,17].

**Figure A1:**
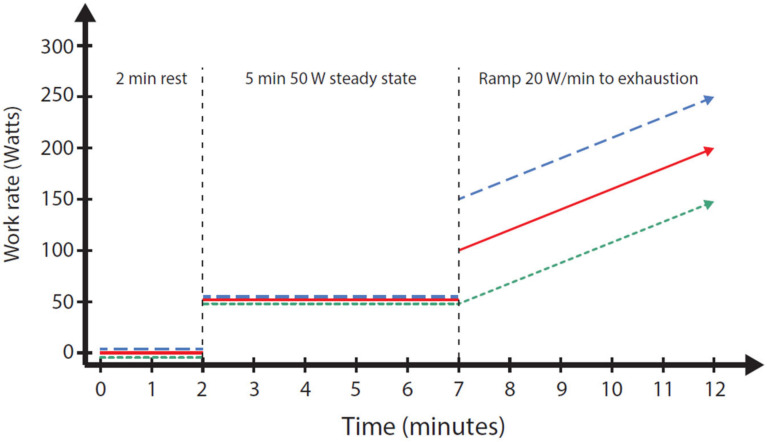
Illustration of the three different exercise protocols used during the incremental bicycle ergometer cardiopulmonary exercise test in athletes. After five minutes of the steady state at 50 Watts, the ramp phase commenced at either 50 Watts (green line), 100 Watts (red line) or 150 Watts (blue line) and continued at 20 Watts/min until exhaustion.

The participants were instructed to refrain from strenuous exercise at least 24 h prior to the cardiopulmonary exercise test (CPET) and from caffeine two hours prior to the test. Maximal CPET was performed on an electronically braked cycle ergometer (eBike Comfort, Ergoline GmbH, Bitz, Germany) using an individualized ramp protocol aiming for a total ramp phase of 8–12 min. Two minutes of rest sitting on the ergometer was followed by five minutes of warmup at 50 W. The work rate then increased instantly to 50 W, 100 W or 150 W depending on the subject’s expected maximal work capacity, followed by a continuous increase by 20 W/min (Appendix A Figure A1) until as near the maximal exertion as possible, aiming for a plateau in VO_2_ and/or a respiratory exchange ratio (RER) > 1.1. VO_2max_ was defined as the mean of the two highest consecutive 10 s averages at the end of exercise.

Ventilation and gas exchange parameters (VO_2_ and carbon dioxide elimination (VCO_2_)) were measured breath-by-breath throughout the test (Vyntus CPX, Carefusion GmbH, Hoechberg, Germany). Continuous ECG recordings and heart rate monitoring were made from a few minutes before the start of the exercise until the last blood pressure measurement ten minutes after the test (CardioSoft version 6.73, GE Medical Systems, Milwaukee, WI, USA). The participants were asked to rate their level of perceived exertion (Borg RPE scale) and dyspnea (Borg CR10 scale) every second minute during the test.

SBP and diastolic blood pressure (DBP) were measured in the supine position before the start of the test. During incremental exercise, SBP was measured every second minute, and also specifically at the end of the 5 min warmup (50 W), at 200 W and at peak exercise. If no SBP measurement was made during the last two minutes before test termination, the SBP_max_ was considered as missing. For each measurement during exercise, the subjects were asked to relax and extend the right arm while SBP was measured using a manual sphygmomanometer and a Doppler probe placed over the radial artery.

The maximal work rate (W_max_) and VO_2max_ were compared to the predicted reference values [18,19]. MET was calculated as VO_2_ (mL/kg/min)/3.5 [20]. 

Three different measurements of the work rate-indexed SBP response during exercise were calculated. First, the SBP/W slope was calculated as the difference in SBP between the first and the last SBP measurement during exercise divided by the increment in W between these measurements [9]. Second, using the same rationale, the SBP/VO_2_ slope was calculated by dividing the increment in SBP by the difference in VO_2_, i.e., with data from the timepoints for the first and the last SBP measurement during exercise. Third, the SBP/MET slope was calculated using two different methods: (a) by using the same datapoints as for the SBP/VO_2_ slope (above) and (b) by replacing the first SBP measure during exercise with SBP at rest, in the sitting position, and using one MET as oxygen uptake at rest to allow for comparison with previous studies [10,11,21]. Predicted values for the SBP_max_ and the SBP/W slope were calculated using the formulas provided by Hedman et al. [9].

All data are presented as the means ± standard deviation (SD) or as the median and interquartile range (IQR) based on the distribution. Distribution of data was evaluated by the Shapiro–Wilk test. Paired-samples t-test was used to compare subjects’ actual versus predicted SBP_max_ and SBP/W slope values. Men and women were compared with the independent-samples t-test and the Mann–Whitney U-test, depending on the distribution of data. Correlations between the variables were explored using the Pearson r. Statistical analyses were performed using SPSS statistics software version 27.0 (IBM Corp., Armonk, NY, USA). Two-sided statistical significance was set at *p* < 0.05 in all the analyses. 

## 3. Results

### 3.1. Cohort Characteristics

A total of 27 subjects (16 men) were included (Table 1). There were no current smokers, but three subjects reported previous smoking (all > 15 years ago), seven subjects reported use of any medication (anti-asthmatic drugs, *n* = 4; other non-cardiovascular drugs, *n* = 3).

The male athletes reached higher absolute work rate and VO_2max_, whereas body weight-indexed oxygen uptake (mL/kg/min) did not differ significantly between the men and the women. Female athletes achieved a higher % of the predicted work rate and a higher % of the predicted VO_2max_ than the men. 

### 3.2. Blood Pressure Response to Exercise and Correlation to the Maximal Oxygen Uptake

An SBP measurement during the last two minutes of exercise (SBP_max_) was missing in six men and one woman, and they were excluded from analyses regarding the SBP_max_ (but were included in analyses of the slopes). The men reached a higher absolute SBP_max_ than women, while there were no differences at fixed work rates (50 and 200 W). The mean SBP/W slope was 0.27 ± 0.08 mm Hg/W, and the mean SBP/VO_2_ slope was 29.8 ± 10.2 mm Hg/L/min, similar between the men and the women (Table 2). 

**Figure 1:**
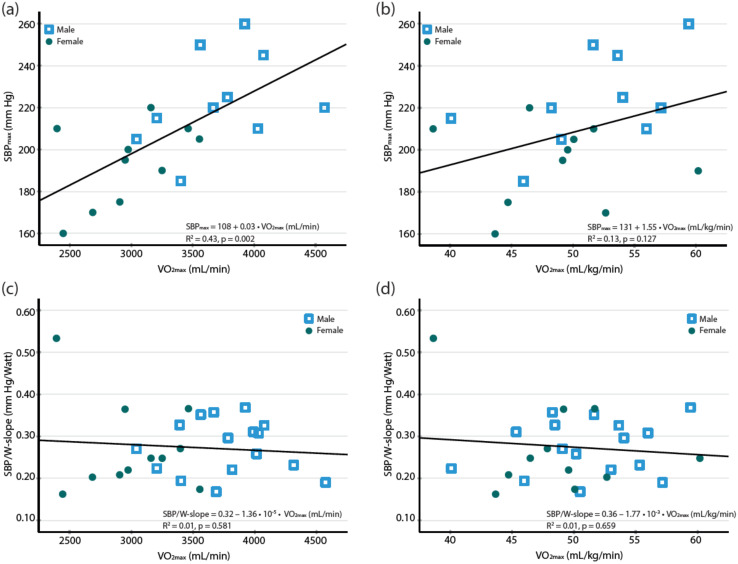
Relation between the maximal oxygen uptake (VO_2max_) and blood pressure response (SBP) to exercise in endurance athletes described as (**a**) VO_2max_ (mL/min) and SBP_max_, (**b**) VO_2max_ (mL/kg/min) and SBP_max_, (**c**) VO_2max_ (mL/min) and SBP/W slope, (**d**) VO_2max_ (mL/kg/min) and SBP/W slope.

Absolute VO_2max_ (mL/min) was correlated to the SBP_max_ (Figure 1) but not to any of the SBP measures indexed to work rate (SBP/W slope) or to VO_2_ (SBP/VO_2_ slope and SBP/MET slope) during exercise (Table 3). Body weight-indexed VO_2max_ (mL/kg/min) was not correlated to neither SBP_max_ nor to any of the SBP measures indexed to work rate or VO_2_ (Figure 1, Table 3). 

As seen in Figure 1, one female athlete was an outlier in regards of the SBP/VO_2_ slope, the SBP/MET slope and the SBP/W slope. Detailed examination of echocardiography and CPET revealed no abnormalities. The outlier had only minor effects on the mean and median values of the SBP measures (Appendix A Table A1). 

### 3.3. Comparison with the Suggested Thresholds and Reference Equations

In 21 out of the 27 athletes (78%), the peak systolic blood pressure was exaggerated according to the fixed thresholds suggested in the current guidelines (210 mm Hg in men and 190 mm Hg in women) [1], and the mean SBP_max_ in both male and female athletes exceeded these sex-specific thresholds (Table 2).

**Figure 2:**
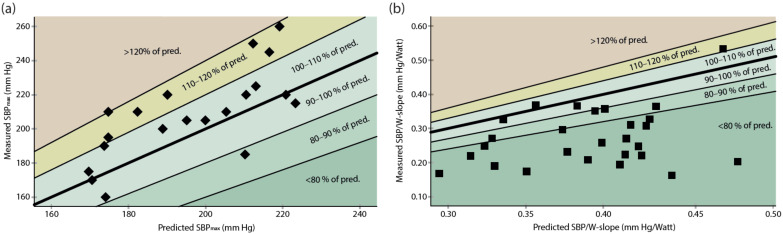
Measured exercise blood pressure in the athletes compared to the predicted values from the reference equation. Each black square represents the observation from an individual athlete. Reference line represents a correlation of 1.0. (**a**) Maximal systolic exercise blood pressure (SBP_max_) versus predicted SBP_max_ (*n* = 20). (**b**) Measured SBP/W slope versus predicted SBP/W slope (*n* = 27).

In the athletes, the SBP_max_ was significantly higher than predicted by the reference equation for the general population (mean difference, 12.2 ± 17.6 mm Hg; 95% confidence interval (CI), 4.0–20.5) (Figure 2). In the female athletes, the mean SBP_max_ was higher than predicted by the reference equation (mean difference, 14.1 ± 15.0 mm Hg; 95% CI, 3.3–24.9). The mean difference in the measured minus predicted SBP_max_ in the men was 10.4 ± 20.6 mm Hg (95% Cim −4.3–25.1). 

In contrast, the mean value of the SBP/W slope was lower than predicted by the reference equation (mean difference, −0.12 ± 0.08 mm Hg/W; 95% CI, −0.08–−0.15), significantly so also when the men and the women were analyzed separately (in the men, mean difference, −0.11 ± 0.07 mm Hg/W, 95% CI, −0.15–−0.08; in the women, −0.12 ± 0.11 mm Hg/W, 95% CI, −0.19 – −0.05) (Figure 2).

## 4. Discussion

We showed that in endurance athletes achieving a high work rate (W_max_) and with a high aerobic capacity (VO_2max_), the SBP_max_ was higher than predicted using a recent reference equation for the general population [9]. In contrast, the work rate-indexed SBP response (SBP/W slope) was lower in athletes than predicted. Although the SBP_max_ was higher in male than in female athletes, when work rate was considered (SBP/W slope), there was no difference in the SBP response to exercise between male and female athletes. This and the fact that 78% of the athletes exceeded the suggested thresholds for an exaggerated blood pressure response to exercise [2] underscore the importance of considering work rate in evaluation of exercise SBP in athletes as well as of considering specific reference equations when interpreting the SBP response in athletes.

During pre-participation evaluation of athletes, resting SBP has been found to exceed the threshold for hypertension (140 mm Hg) in about 3–8% of examined athletes [22,23], recognizing a need to account for hypertension in the athletic population as well. Although athletes often undergo exercise testing during their career, the SBP response to exercise in athletes has not gained attention until recently [6], and data on the work rate-indexed SBP blood pressure response in athletes are scarce [11,12,21]. Ultimately, better understanding of the blood pressure physiology in athletes could guide physicians in discriminating a pathological blood pressure response, possibly implying an increased risk of hypertension [24] or other cardiovascular disease, from a physiologically high exercise SBP. 

In the present study, the absolute VO_2max_ (mL/min) was significantly correlated to the SBP_max_, as previously shown [12], which supports that a high SBP_max_ during exercise in athletes can reflect a high fitness level [25] and be related to the increased CO during high work rates. However, we found no significant correlations between the weight-adjusted maximal oxygen uptake and the SBP/VO_2_ slope. One possible reason might be that we only studied relatively fit subjects within a narrow span of the body weight indexed VO_2max_, and it is possible that with a greater span of fitness level, correlations would be present. Neither did we find any significant correlations between the VO_2max_ (mL/min or mL/kg/min) and the SBP/W slope. 

Comparison across previous studies on the work rate indexed SBP response in athletes is somewhat limited by the use of different exercise test protocols. Our cycle ergometry test protocol included a rapid increase from the warmup at 50 W to either 100 W or 150 W just before starting the continuous work rate increment for most participants. This attempt to optimize the total exercise time may have influenced the SBP measurements for some subjects, especially those with a relatively low maximal work rate. Further, cycling ergometry has been shown to elicit higher SBP recordings compared to treadmill testing [26], at least in men [12], which limits comparisons across studies employing different testing modalities. However, a few meaningful comparisons can be made. 

We found lower SBP/W slope values than previously reported in a sample of 95 professional male German handball players [11]. Although they were younger, the maximal work rate of the handball players was similar to that of the male athletes in the present study. Their SBP_max_ was lower (mean, 200 mm Hg), but they had a higher SBP/W slope of 0.35 mm Hg/W compared to what was found in the present study (Table 4). A higher SBP_max_ in our study sample was expected considering population-based data of increasing SBP_max_ with increasing age [9], whereas the lower SBP/W slope found in the present study was somewhat unexpected based on the fact that the SBP/W slope also increases with age, at least in the general population [9]. Additional factors may also have influenced the different SBP/W slopes, such as different exercise protocols and the fact that different training regimes used by endurance athletes compared to athletes in team sports may affect the blood pressure response to exercise. Future studies may reveal if and how the work rate indexed SBP response to exercise differs between different sports disciplines. Of note, in the same study by Bauer et al., the SBP at 200 W was measured, with markedly lower mean values than those of the male endurance athletes included in the present study (Table 4).

We found no significant difference in the SBP/VO_2_ slope between the male and female endurance athletes, as opposed to Petek et al. who found a significantly higher SBP/VO_2_ slope in female athletes [12]. The absent sex difference in the SBP/VO_2_ slope in our sample of athletes might have been affected by the female athletes having a relatively higher level of fitness as compared to our male athletes (VO_2max_, 169% vs. 146% of the predicted values, *p* < 0.001). However, the difference in the mean age between the men and the women in our sample might also have somewhat limited the comparison to previous studies (Table 4).

A key finding was that the majority of athletes reached SBP values exceeding the proposed upper limits for normal peak SBP. Although sometimes neglected in clinical practice, work rate has a direct impact on exercise SBP, which challenges the clinical interpretation of a high peak SBP as pathological or physiological. Since high SBP_max_ has been associated with an increased risk of developing hypertension at rest in the general population [24,27] as well as in young athletes [6], it is plausible that blood pressure slope values, taking into account work rate and fitness, may be a better predictor of the future risk of hypertension in this group. This, however, remains to be evaluated.

Another implication of the present findings is that the SBP/VO_2_ slope provides a novel, precise way of indexing the SBP response to work rate and could provide a valuable tool in further studies investigating the SBP response to exercise. The SBP/VO_2_ slope has been only scarcely studied in athletes previously, and normative values are lacking. In our sample of endurance athletes, the mean SBP/VO_2_ slope was 30 mm Hg/L/min, which is slightly higher than the values recently reported by Petek et al. [12] (Table 4). The slope calculated from low work rate in our study, compared to that from sitting rest used by Petek et al., may have contributed to the difference. In addition, as we found a correlation between the absolute VO_2 max_ (mL/min) and the SBP_max_, the higher fitness among the athletes in our study sample suggests a need for additional studies of athletes at different fitness levels to better understand the physiology of SBP response in relation to VO_2_. 

The strength of the present study is a contribution to the relatively novel field of exercise SBP in athletes, in particular in relation to work rate and VO_2_. The limitation is the relatively small sample size. Although we believe our results give a framework for the range of SBP measures expected in athletes, they cannot be used as reference values. Second, all the included subjects were well-trained endurance athletes of middle age. Although this complements previously published data in younger athletes of other sport disciplines [10,21], our results should not be extrapolated to athletes of all ages and all sports. Third, our CPET protocol included an instant increase in work rate at the start of the ramp protocol in order to reduce the total work time for athletes while allowing a standardized measure at 50 W for all subjects. This rapid increase in work rate may theoretically have influenced the results for some of the participants, especially for those reaching a relatively low maximal work rate.

## 5. Conclusions

Endurance athletes of middle age had higher SBP_max_ values, the majority exceeding the proposed upper limits for normal peak SBP, but a lower SBP/W slope than predicted using reference equations for the general population. The SPB_max_ correlated to the absolute VO2_max_, but there was no correlation between the VO_2max_ (mL/kg/min or mL/min) and the measurements of the work rate-indexed blood pressure response to exercise. Our results indicate that different reference equations than in the general population might be needed for the SBP response in athletes to assist in discrimination between physiologically high and pathologically high systolic blood pressure responses. Considering the small sample size, this needs to be confirmed in larger studies as well as in athletes of varying age.

## Figures and Tables

**Table 1:** Subject characteristics and cardiopulmonary exercise test data.

	All Subjects	Men	Women	*p*
	(*n* = 27)	(*n* = 16)	(*n* = 11)	
**Demographics**				
Age (years)	40 ± 10	45 ± 10	33 ± 6	0.002
Weight (kg)	69 ± 9	74 ± 6	62 ± 7	<0.001
Height (cm)	176 ± 8	180 ± 7	170 ± 5	<0.001
BMI (kg/m^2^)	22 ± 2	23 ± 1	22 ± 2	0.045
**Cardiopulmonary exercise test**				
VO_2max_ (L/min)	3.5 ± 0.5	3.9 ± 0.4	3.0 ± 0.4	<0.001
VO_2max_ (mL/kg/min)	50 ± 5	51 ± 5	49 ± 6	0.23
VO_2max_ (% of predicted)	155 ± 19	146 ± 13	169 ± 17	<0.001
MET_max_	14 ± 2	15 ± 1	14 ± 2	0.23
RPE_max_ (Borg RPE)	19 (2)	19 (2)	17 (2)	0.09
Dyspnea_max_ (Borg CR10)	8 (2)	9 (1)	7 (2)	0.034
HR_max_ (beats/min)	175 ± 10	175 ± 12	175 ± 9	0.89
HR_max_ (% of age-predicted)	97 ± 6	100 ± 6	94 ± 4	0.008
W_max_	315 ± 58	349 ± 42	266 ± 37	<0.001
W_max_ (% of predicted)	154 ± 19	146 ± 17	166 ± 16	0.004

Data presented as the means ± standard deviation except for RPE_max_ and Dyspnea_max_, which are presented as the medians (interquartile range). *p*-value for comparison between the men and the women. BMI: body mass index; RPE: rating of perceived exertion; CR10: category ratio; HR: heart rate; W: Watt; VO_2_: oxygen uptake; MET: metabolic equivalents of task; _max_: highest value during the test.

**Table 2:** Blood pressure data.

	All subjects	Men	Women	*p*
	(*n* = 27)	(*n* = 16)	(*n* = 11)	
**At rest, before the test**				
SBP_lying_ (mm Hg)	124 ± 12	128 ± 10	118 ± 11	0.022
DBP_lying_ (mm Hg)	75 ± 8	75 ± 7	74 ± 8	0.68
SBP_sitting_ (mm Hg)	125 ± 14	130 ± 11	118 ± 13	0.025
**During exercise**				
SBP_max_ (mm Hg) ^1^	209 ± 26	224 ± 23	194 ± 20	0.005
SBP_peak_ (mm Hg)	211 ± 24	223 ± 20	193 ± 19	<0.001
SBP_200 W_ (mm Hg)	188 ± 18	190 ± 17	184 ± 20	0.35
SBP_50 W_ (mm Hg)	145 ± 15	148 ± 15	140 ± 16	0.16
**Indexed to exercise intensity or work rate**				
SBP/VO_2_ slope (mm Hg/L/min)	29.8 ± 10.2	29.5 ± 6.4	30.3 ± 14.5	0.85
SBP/MET slope (mm Hg/MET) ^2^	7.2 ± 2.4	7.6 ± 1.6	6.6 ± 3.3	0.30
SBP/MET slope (mm Hg/MET) ^3^	6.8 ± 1.3	7.2 ± 1.0	6.3 ± 1.6	0.070
SBP/W slope (mm Hg/W)	0.27 ± 0.08	0.28 ± 0.06	0.27 ± 0.11	0.94

Data presented as the means ± standard deviation. *p*-value for comparison between the men and the women. ^1^ Data regarding the SBP_max_ include only the subjects with valid measurements within 2 min before the end of exercise (*n* = 20, 10 men and 10 women) ^2^ Calculated from the steady state at 50 W to the last SBP measurement during exercise. ^3^ Calculated from resting SBP (MET = 1) to last the SBP measurement during exercise. Data for one woman were missing regarding resting SBP and DBP measured in the supine position. SBP: systolic blood pressure; DBP: diastolic blood pressure; VO_2_: oxygen uptake; MET: metabolic equivalents of task; W: Watt; SBP_50 W_: SBP measured at 50 W; SBP_200 W_: SBP measured at 200 W; SBP_max_: SBP within 2 min before the end of exercise; SBP_peak_: highest measured SBP during exercise.

**Table 3:** Correlation between exercise systolic blood pressure (SBP) measures and the maximal oxygen uptake (VO_2max_) expressed as both the absolute value (mL/min) and the body weight-indexed (mL/kg/min) value.

	VO_2max_ (mL/min)	VO_2max_ (mL/kg/min)
SBP_max_^1^, r (*p*)	0.654 (0.002)	0.353 (0.13)
SBP/VO_2_ slope, r (*p*)	−0.205 (0.31)	−0.258 (0.19)
SBP/MET slope ^2^, r (*p*)	0.066 (0.75)	−0.266 (0.18)
SBP/MET slope ^3^, r (*p*)	0.140 (0.49)	−0.290 (0.14)
SBP/W slope, r (*p*)	−0.089 (0.66)	−0.111 (0.58)
SBP at 50 W, r (*p*)	0.156 (0.44)	0.005 (0.98)
SBP at 200 W, r (*p*)	0.048 (0.81)	−0.050 (0.81)

^1^ Calculated for subjects with SBP measurements within the last 2 min of exercise, *n* = 20. ^2^ Calculated from the steady state at 50 W to the last SBP measurement during exercise. ^3^ Calculated from resting SBP (MET = 1) to the last SBP measurement during exercise.

**Table 4:** Comparison of subject characteristics and bicycle cardiopulmonary exercise test results in the current and previous studies on the systolic blood pressure response to exercise in athletes.

	Current Study		Bauer et al.			Petek et al.	
			[11]	[10]		[12]	
	Male endurance athletes	Female endurance athletes	Male handball players	Male handball players	Female football players	Male athletes	Female athletes
Age (years)	45 ± 10	33 ± 7	26 ± 5	22 ± 2	21 ± 2	38 ± 16	34 ± 15
Height (cm)	179 ± 7	170 ± 6	189 ± 7	189 ± 7	167 ± 5	NA	NA
Weight (kg)	73.7 ± 6.4	62.9 ± 6.9	91.5 ± 10.7	90.9 ± 12.3	60.8 ± 7.7	NA	NA
BMI (kg/m^2^)	22.9 ± 1.4	21.8 ± 1.8	25.7 ± 2	25.5 ± 2.4	21.7 ± 1.9	NA	NA
**Before the test, at rest**							
SBP_sitting_ (mm Hg)	130 ± 12	120 ± 14	123 ± 10	125 ± 10	120 ± 11	121 ± 9	110 ± 10
**At peak exercise**							
W_max_	344 ± 39	269 ± 39	339 ± 64	342 ± 72	190 ± 32	344 ± 72	211 ± 48
VO_2max_ (mL/kg/min)	51 ± 5	49 ± 6	NA	NA	NA	46 ± 10	37 ± 8
HR_max_ (beats/min)	174 ± 12	176 ± 9	179 ± 10	179 ± 12	184 ± 8	175 ± 16	175 ± 15
HR_max_ (% predicted)	100 ± 6	94 ± 3	94 ± 5	NA	NA	NA	NA
**SBP response to exercise**							
SBP_max_ (mm Hg)	224 ± 24	194 ± 20	200 ± 20	202 ± 20	177 ± 15	186 ± 24	161 ± 15
SBP/W slope (mm Hg/W)	0.28 ± 0.06	0.27 ± 0.11	0.34 ± 0.13	0.34 ± 0.12	0.53 ± 0.19	0.20 ± 0.06	0.25 ± 0.08
SBP_200 W_ (mm Hg)	191 ± 17	184 ± 22	169 ± 18	NA	NA	NA	NA
SBP/VO_2_ slope (mm Hg/L/min)	30 ± 6	30 ± 15	NA	NA	NA	21 ± 7	26 ± 7

Data presented as the means ± standard deviation. BMI: body mass index; SBP: systolic blood pressure; RPE: rating of perceived exertion; HR: heart rate; W: Watt; _sitting_: sitting on a bicycle before exercise; _max_: highest value during the test; _200 W_: measured at 200 W.

## Data Availability

Data available on reasonable request.

## Appendix A

**Table A1:** One female SBP outlier was identified. This table presents the effect on the data when the outlier was excluded.

	Outlier Included	Outlier Excluded
SBP/VO_2_ slope in all athletes, mm Hg/L/min	30.0 ± 10.2	28.4 ± 6.9
SBP/VO_2_ slope in female athletes, mm Hg/L/min	30.3 ± 14.5	26.5 ± 7.7
SBP/W slope in all athletes, mm Hg	0.27 ± 0.08	0.26 ± 0.07
SBP/W slope in female athletes, mm Hg	0.27 ± 0.11	0.25 ± 0.07

SBP: systolic blood pressure; VO_2_: oxygen uptake; W: Watt.
